# Short-Term Intensified Cycle Training Alters Acute and Chronic Responses of PGC1α and Cytochrome C Oxidase IV to Exercise in Human Skeletal Muscle

**DOI:** 10.1371/journal.pone.0053080

**Published:** 2012-12-28

**Authors:** Nigel K. Stepto, Boubacar Benziane, Glenn D. Wadley, Alexander V. Chibalin, Benedict J. Canny, Nir Eynon, Glenn K. McConell

**Affiliations:** 1 Institute of Sport Exercise and Active Living, Victoria University, Footscray, Victoria, Australia; 2 School of Sport and Exercise Science, Victoria University, Footscray, Victoria, Australia; 3 Department of Physiology and Pharmacology, Karolinska Institutet, Stockholm, Sweden; 4 Centre for Physical Activity and Nutrition Research, School of Exercise and Nutrition Sciences, Deakin University, Burwood, Victoria, Australia; 5 Department of Molecular Medicine and Surgery, Karolinska Institutet, Stockholm, Sweden; 6 Department of Physiology Monash University, Clayton, Victoria, Australia; 7 School of Biomedical and Health Sciences, Victoria University, Footscray, Victoria, Australia; University of Texas Health Science Center at San Antonio, United States of America

## Abstract

Reduced activation of exercise responsive signalling pathways have been reported in response to acute exercise after training; however little is known about the adaptive responses of the mitochondria. Accordingly, we investigated changes in mitochondrial gene expression and protein abundance in response to the same acute exercise before and after 10-d of intensive cycle training. Nine untrained, healthy participants (mean±SD; VO_2peak_ 44.1±17.6 ml/kg/min) performed a 60 min bout of cycling exercise at 164±18 W (72% of pre-training VO_2peak_). Muscle biopsies were obtained from the vastus lateralis muscle at rest, immediately and 3 h after exercise. The participants then underwent 10-d of cycle training which included four high-intensity interval training sessions (6×5 min; 90–100% VO_2peak_) and six prolonged moderate-intensity sessions (45–90 min; 75% VO_2peak_). Participants repeated the pre-training exercise trial at the same absolute work load (64% of pre-training VO_2peak_). Muscle PGC1-α mRNA expression was attenuated as it increased by 11- and 4- fold (P<0.001) after exercise pre- and post-training, respectively. PGC1-α protein expression increased 1.5 fold (P<0.05) in response to exercise pre-training with no further increases after the post-training exercise bout. RIP140 protein abundance was responsive to acute exercise only (P<0.01). COXIV mRNA (1.6 fold; P<0.01) and COXIV protein expression (1.5 fold; P<0.05) were increased by training but COXIV protein expression was decreased (20%; P<0.01) by acute exercise pre- and post-training. These findings demonstrate that short-term intensified training promotes increased mitochondrial gene expression and protein abundance. Furthermore, acute indicators of exercise-induced mitochondrial adaptation appear to be blunted in response to exercise at the same absolute intensity following short-term training.

## Introduction

Acute and chronic exercise models have been used to investigate mitochondrial adaptations in skeletal muscle. Traditional studies investigating mitochondrial adaptations were conducted utilising prolonged endurance training protocols in rodents [1], [2] and humans [3]. These and other studies demonstrated that 6–12 weeks of endurance exercise training increased the capacity of the mitochondria to produce ATP. This enhanced function is mediated by increased levels of the mitochondrial proteins that regulate substrate entry and oxidation, the tricarboxylic acid cycle, respiratory chain, and ATP synthesis (reviewed in [4]). Recently, expansion of mitochondrial volume and function have been suggested to increase rapidly with mitochondrial protein content increases observed after as little as six to ten consecutive [5], [6] and non-consecutive [7]–[13] days of training in humans.

The mitochondrial biogenesis transcriptional programme in skeletal muscle results from the coordinated activation of signalling pathways, transcription co-activators, transcription factors, and repressors initiated in response to muscle contraction [14]. Specifically, the activation of AMP-activated protein kinase (AMPK), calcium/calmodulin-dependent protein kinase (CaMK) II and p38 mitogen-activated protein kinase (p38 MAPK) signalling cascades are well characterized upstream modulators of peroxisome proliferator activated receptor γ co-activator -1α (PGC-1α) gene expression in skeletal muscle [15]–[17]. These cascades activate downstream regulatory factors [15], [17], and in the case of AMPK [16] and p38 [18], also directly phosphorylate PGC-1α, thereby increasing transcriptional activation of the PGC-1α. Also, repressors interact with co-activators, such as the receptor interacting protein 140 (RIP 140; [19]) to help regulate mitochondrial biogenesis. RIP140 is subject to post-translational modifications like ubiquitination, acetylation, phosphorylation, and methylation, via a yet to be defined signalling pathways, and a number of these modifications have been shown to affect its function [20], [21].

Given that skeletal muscle energy flux during contraction is effected by exercise training status, it is not surprising that signal transduction cascades are differentially regulated by acute exercise before and after training [22], [23]. Specifically, AMPK and p38 MAPK phosphorylation are attenuated in response to the same exercise bout after training [22] or in a highly trained compared to untrained individuals [24]. Furthermore, exercise intensity and/or total work done not only determine signalling cascade activation but also gene expression of genes like PGC1α [25], [26]. However, it is unknown whether this training-induced reduction in relative exercise intensity and reduced activation of these signalling cascades lead to down regulation of target genes (for example PGC1α,β, PGC-1 related co-activator [PRC], mitochondrial transcription factor A [Tfam] and nuclear respiratory factor 1 [NRF1]) or alterations in transcriptional regulation by PGC1α and RIP140 proteins that determine exercise induced mitochondrial reticulum expansion.

At present, there remains a significant gap in the literature on the molecular events (gene expression and changes in protein abundance) that occur in the process of mitochondrial biogenesis and reticulum expansion in human skeletal muscle in response to acute exercise before and after intensified endurance exercise training. The purposes of this study were to firstly examine changes in expression of mitochondrial genes that are involved in the regulation of mitochondrial biogenesis (PGC-1α and β, PRC, Tfam, NRF 2) under exercise conditions that maximally highlighted training adaptions (exercise at the same absolute intensity). Secondly, to investigate changes in protein abundance of mitochondrial biogenesis regulators and mitochondrial function including PGC-1α, RIP140 and the Electron Transport System (ETS) proteins. We hypothesized that with exercise training there would be an attenuation of the increase in key mitochondrial genes expressed in response to the same absolute bout of exercise before compared with after training. Additionally, we hypothesized that there will be an increase in mitochondrial protein abundance after training with no effects of acute exercise both before and after training.

## Materials and Methods

### Ethics Statement

The study protocols were approved by the Monash University Standing Committee on Ethics in Research Involving Humans and the Karolinska Institutet Human Research Ethics Committee, and conducted according to the principles expressed in the Declaration of Helsinki. All participants were fully informed of the possible risks involved in the study before providing written informed consent.

### Participants

Nine healthy untrained male volunteers were recruited for the short-term training study (age: 23±9 year; weight: 79±24 kg; height: 179±24 cm; VO_2peak_ 3.5±1.8 l/min). The data from this study forms part of a larger study investigating the molecular adaptations to short-term intensified cycle training in humans and have previously been reported [22].

### Study Design

**Figure 1:**
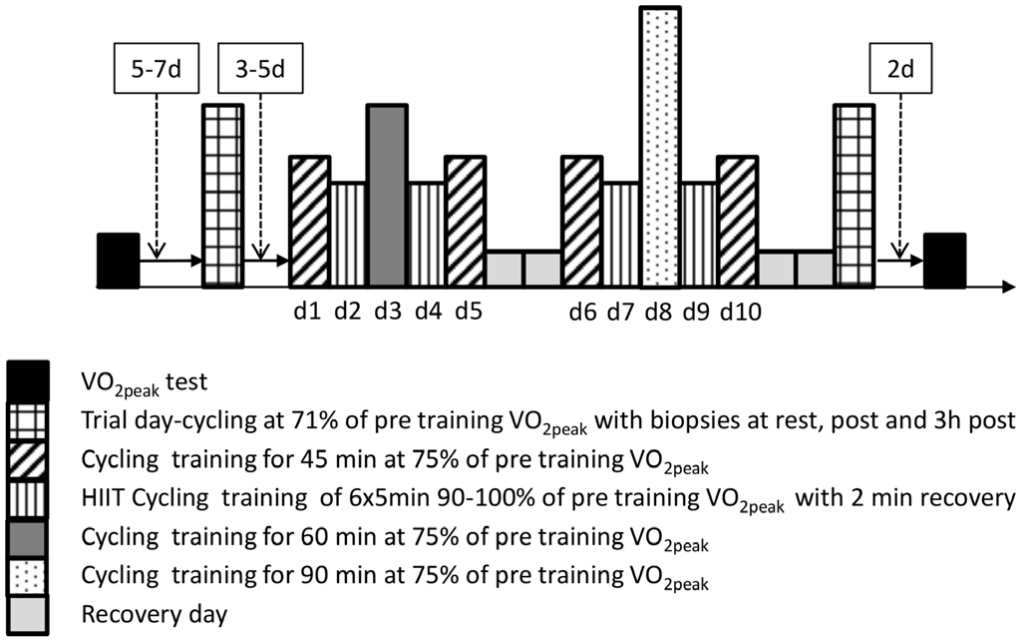
Schema of the study design showing the 4 testing and 10 training sessions undertaken by each participant. D-day; HIIT-High-intensity interval-training; VO_2peak_-maximal oxygen consumption.

The study design (Figure 1) has previously been reported [22]. Briefly, to investigate the effects of training on molecular markers of mitochondrial biogenesis and reticulum expansion we utilized a short-term training protocol that improved exercise performance, altered muscle metabolism and cell signaling [22], [23]. Participants completed a cycling VO_2 peak_ test on the Lode cycle ergometer 5–7-days prior to the pre-training acute exercise bout. The 10-day cycle training regimen commenced approximately one week later and consisted of 10 days of endurance cycle training. The training programme included 4 days of high intensity interval training, and six sessions of varying durations of moderate intensity cycling (Figure 1). After two days of recovery the second acute exercise trial was undertaken at the same absolute workload as the pre-training trial. Skeletal muscle biopsies were taken at rest, immediately and 3 h post exercise from the vastus lateralis, while blood and expiratory gases were taken during these acute exercise trials [22].

### Dietary and Exercise Control

Subjects were instructed to abstain from caffeinated products and alcoholic beverages 24 h prior to the exercise trials while consuming their normal diet, which they recorded in daily food diaries during the 3 days prior to the exercise trials. They were instructed to consume the same food or record any changes from the initial diet. This dietary control was effective, as diet and energy consumption was unchanged (10.6±1.6 MJ/d pre- and post-training) [22].

### Pre- and Post-exercise Trials

Subjects reported to the laboratory between 0600 or 0700 hours after an overnight fast (∼9 h). Upon arrival, subjects voided and body weight was measured. After 15 min of supine rest the first of three muscle biopsies (120–250 mg each) was obtained, after administration of local anaesthesia, the remaining biopsies were obtained from separate incision sites over the *vastus lateralis*. Following the initial biopsy, subjects rode at 71±8% and 64±9% VO_2 peak_ (164±18 W) for 60 min pre- and post-training respectively. The second muscle biopsy was taken immediately (<20 sec) after the completion of the ride. Subjects then rested in a supine position, and were instructed to keep as still as possible for the next 3 h after which the final biopsy was taken from the most proximal incision. Drinking water was provided *ad libitum* to subjects to consume over the duration of the trial but matched for post training trial. Muscle biopsies were immediately frozen in liquid nitrogen and stored at −80°C until analysis. All metabolic measures, muscle glycogen and selected cell signaling data for these subjects have been previously reported [22].

### Antibodies and Reagents

All antibodies were purchased from commercial suppliers. The antibodies use were against total NADH-ubiquinol oxidoreductase (NUO-[complex (C)-I]; Molecular Probes A21344; 1∶5000.) ubiquinol cytochrome c oxidoreductase (Core1 [C-III]; Molecular probes A21362; 1∶7500), Succinate Ubiqinone Oxyreductase (SUO [C-II]; Molecular probes A11142; 1∶20000); Cytochrome C Oxidase I (COXI [C-IV]; Molecular probes A21344; 1∶5000) and Cytochrome C Oxidase IV (COXIV [C-IV]; Abcam ab14744 1:1000), PGC1α (peroxisome proliferator-activated receptor γ co-activator α; Calbiochem # KP9803;1∶1000), nuclear co-repressor receptor interfering protein 140 kDa (RIP140; Abcam #ab3425 1:1000) and GAPDH (Santa Cruz #sc 25778; 1∶2000). All other reagents were of analytical grade (Sigma, St. Louis, MO), unless otherwise specified.

### RNA Extraction and RT-QPCR

Total RNA was extracted from 30–40 mg of muscle as previously described [27] Briefly, samples were homogenized in 0.5 ml of Trizol reagent (Invitrogen Life Technologies, Australia), and incubated at room temperature for 5 min. After addition of chloroform (100 µl), samples were incubated for 3 min at room temperature, centrifuged for 15 min (12 000 g, 4°C) and the aqueous phase, containing the RNA, was precipitated with an equal volume of 100% ethanol. Total RNA was further purified through a Qiagen RNeasy Micro-column (Qiagen, Germany) according to manufacturer’s protocol. RNA was resuspended in 10 µL RNase-free water. RNA integrity was verified and the concentration determined on the Experion Automated Electrophoresis System (Bio-Rad Laboratories, NSW, Australia).

Reverse transcription was performed on DNase-treated RNA using AMV Reverse Transcriptase (Promega, Madison, WI) as previously described [28]. Following reverse transcription, the remaining RNA was degraded by treatment with RNase H (Invitrogen) for 20 min at 37°C. The amount of single stranded DNA was then determined in each sample compared to an oligonucleotide standard in an assay using OliGreen reagent (Invitrogen), which was incubated in the dark at 80°C for five min prior to the measurement of fluorescence.

Primer sequences were obtained from gene sequences from GenBank and are shown in Table 1. Primer sequences were validated using BLAST to ensure each primer was homologous with the desired mRNA of human skeletal muscle [28]. Real-time PCR using SYBR® Green chemistry was performed using the Stratagene MX3000P sequence detection system (Agilent Technologies, CA, USA). Samples were subjected to a heat dissociation protocol after the final cycle of PCR to ensure that only one product was detected. The mRNA of each gene was normalized to the absolute cDNA content in each sample using the OliGreen assay [28]. This has previously been shown to be a robust and suitable method of normalization that avoids the many problems associated with “housekeeping genes” [29], [30].

**Table 1:** Primer sequences used for RT-qPCR.

Gene		Sense Primer (5′-3′)	Antisense Primer (5′-3′)
PGC1α	NM_013261.3	tgagagggccaagcaaag	ataaatcacacggcgctctt
PGC1β	NM_133263	ctgctggcccagatacactga	atccatggcttcatacttgctttt
PRC	NM_015062.3	gaggattttgggagccttg	gtgagcagcgacacttcatt
TFAM	NM_003201.1	gaacaactacccatatttaaagctca	gaatcaggaagttccctcca
NRF2α	NM_002040.3	actccagccatgactaaaagaga	ggcgcgtaggtttgttctac
COX IV	NM_001861.2	caccgcgctcgttatcat	tggccacccactctttgt
Cytochrome c	NM_018947.4	ggctgcagtgtagctgtgat	gatggagtttcctttatctgttgc
Citrate Synthase	NM_004077.2	gcatcttgtcttgttcttgcag	tggcctgctccttaggtatc
β-HAD	NM_005327.2	ctcggccaagaagataatcg	tctaccaacactactgtgtgacca

### Muscle Protein Extraction and Immunoblot Analysis

Muscle protein extracts were prepared from freeze-dried muscle and the protein concentration of the resulting supernatant was determined using a commercial kit (Bio-Rad, Richmond, CA) as previously described [22]. Aliquots of the muscle lysates were diluted with Laemmli sample buffer, warmed at 56°C for 20 min, and 40 µg of total protein/sample were separated by SDS-PAGE using gradient (6.5–20%) gels. Following electrophoresis, proteins were transferred to polyvinylidene difluoride membranes (PVDF; Millipore, Bedford, MA). Membranes were blocked in 10 mM Tris, 100 mM NaCl, and 0.02% Tween 20 (TBST) containing 5% nonfat milk for 2 h at room temperature, washed with TBST, and then incubated with the appropriate primary antibody overnight at 4°C. Membranes were washed 5 times with TBST, probed with an appropriate horseradish peroxidase-conjugated secondary antibody, according to standard procedures. Proteins were visualized by enhanced chemiluminescence detection (Amersham, Arlington, IL) following the manufacturer's instructions. In order to minimise variability between gels large format gels were used and contain all time points for each participant to reduce the number of gels used. Gels were run and transferred, and the membranes processed at the same time using same solutions of antibodies, washing buffers and chemiluminescence detection. These procedures resulted in no differences between gels in protein loading as confirmed by the determination of mean lane loading from Ponceau staining of the membranes [31]. This low variability was also confirmed by lack of change in abundance of GAPDH across all membranes and time points (data not shown).

### Statistical Analysis

Data is presented as mean ±SD for nine subjects unless otherwise stated. Statistical analysis of gene expression and protein abundance were conducted using two way repeated measures analysis of variance (ANOVA; training x time). When significant interactions were observed a LSD Post-hoc tests were conducted to determine where specific differences occurred. The results were considered significant when p<0.05. Analysis was conducted using the statistical package IBM SPSS Statistics 20.0 for Windows (SPSS, IL, USA).

## Results

### Gene Expression

#### Transcription factors and co-factors

**Figure 2:**
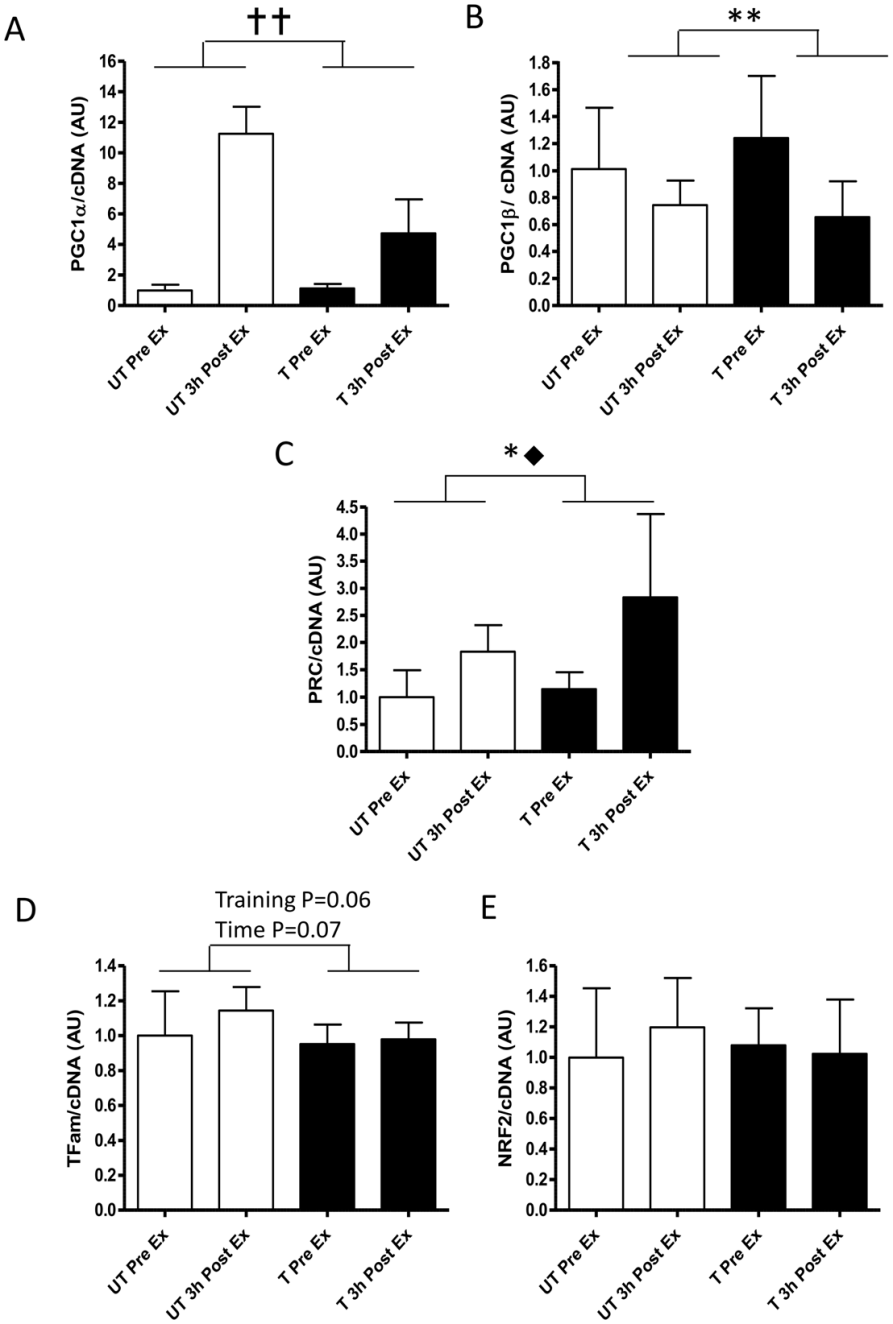
Changes in normalised gene expression of mitochondrial biogenic transcription factors and co-activators in response to the same 1 h cycling bout (∼164W or ∼72% VO_2peak_) before and after 10-d of intensified cycle training. A- peroxisome proliferator-activated receptor γ co-activator (PGC)-1α, B-PGC-1β, C- PGC-1 related co-activator (PRC), D- mitochondrial transcription factor A (Tfam) and E-nuclear respiratory factor 2 (NRF2). Data are mean ±SD for n = 9. pre-training exercise bout (open bars); post-training exercise bout (closed bars); Pre Ex-resting sample just prior to exercise. 3 h Post Ex -sample take 3 h after exercise; 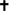 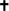
- Training, time and training x time effect P<0.001; **- training x time interaction P<0.05; * - main effect of training P<0.05; ⧫-main effect of time P<0.01.

Acute exercise and exercise training altered the gene expression of the PGC1 co-factor family of genes (Figure 2). PGC1 α mRNA was increase 11.1±3.6 and 4.3±1.2 fold 3 h after exercise pre- and post-training respectively (Training × time P = 0.001; Time P<0.0001; Training P<0.0001; Figure 2A ). In contrast, PGC1β mRNA decreased in response to exercise pre- and- post training by 26±12% and 54±12%, respectively (Training × time P = 0.038; Time P = 0.004; Figure 2B). PRC mRNA increased by 1.8±0.6 fold in response to pre-training exercise and 2.3±0.6 fold to post-training exercise (Time P = 0.004; Training P = 0.018; Figure 2C).

Transcription factor Tfam mRNA trended to increase by 10% (1.1±0.3 fold) in response to exercise pre-training but remained unchanged in response to exercise after training (Time P = 0.06; Training P = 0.07; Figure 2D). However, NRF2 mRNA remained unchanged in response to exercise pre- and post-training (Figure 2E).

#### Mitochondial genes

**Figure 3:**
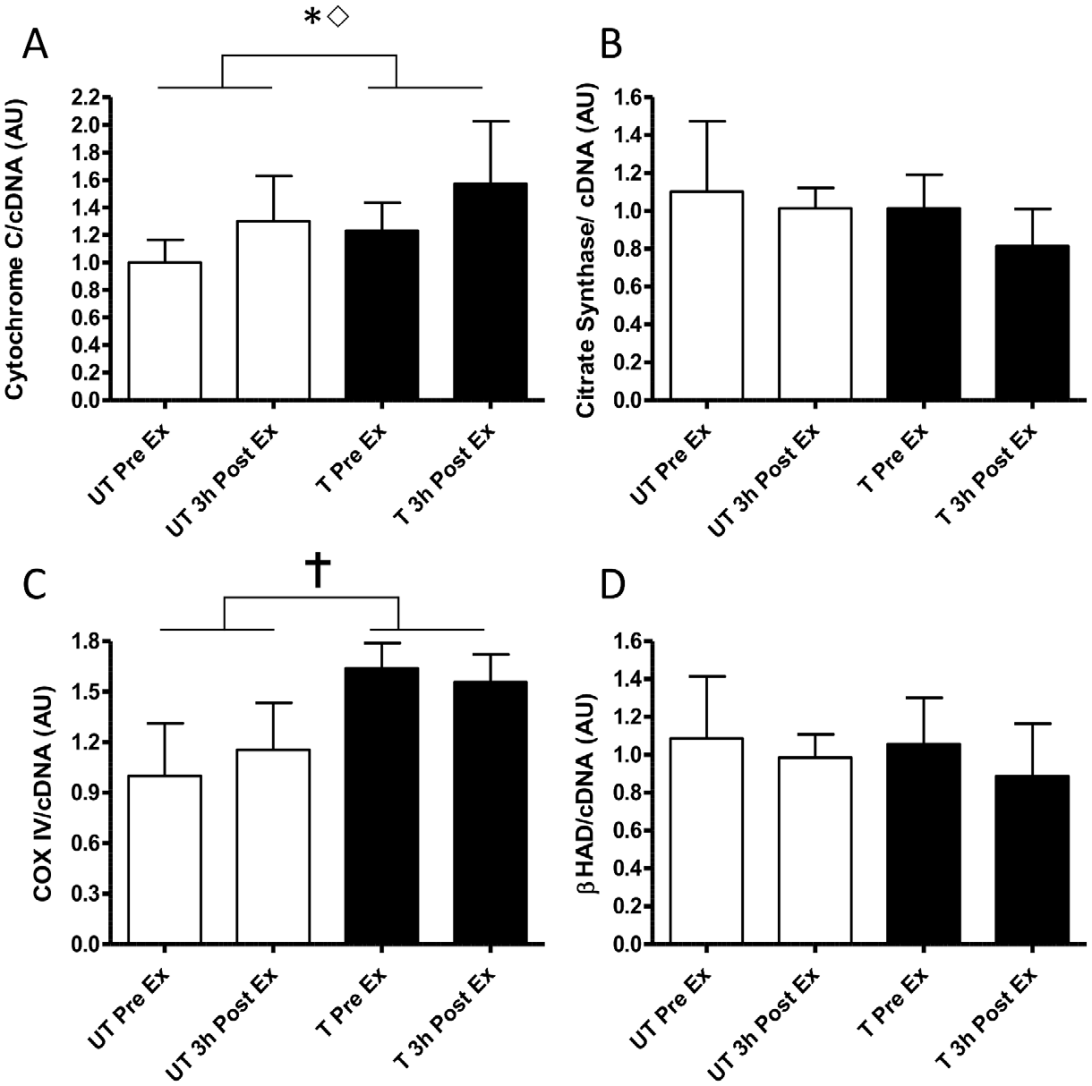
Changes in normalised gene expression of representative mitochondrial proteins in response to the same 1 h cycling bout (∼164W or ∼72% VO_2peak_) before and after 10-d of intensified cycle training. A-,electron transport system protein cytochrome c, B- β-Hydroxyacyl CoA Dehydrogenase (β-HAD), C-cytochrome c oxidase subunit IV (COXIV) and D- citrate synthase. Data are mean ±SD for n = 9. pre-training exercise bout (open bars); post-training exercise bout (closed bars); Pre Ex-resting sample just prior to exercise. 3 h Post Ex -sample take 3 h after exercise; 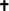
- main effect of training P<0.001; * - main effect of training P<0.05; ⋄-main effect of time P<0.05.

Training increased resting Cytochrome C mRNA by 26% with acute exercise increasing the mRNA (25%–30%) similarly before and after training (1.30±0.24 and 1.25±0.21 fold increase respectively; Figure 3A; training P = 0.018; time P = 0.032). Citrate Synthase (CS) mRNA was neither affected by acute exercise or exercise training (Figure 3B). In contrast, COXIV mRNA was only increased by exercise training (1.6±0.6 fold; Figure 3C; Training P<0.001). β-Hydroxyacyl CoA Dehydrogenase (β-HAD) mRNA trended to a decreased expression in response to acute exercise (3.0±1.2% and 14.0±2.7% before and after training (Figure 3D; time P = 0.07).

### Protein Expression

#### Co- factors and co-repressors

**Figure 4:**
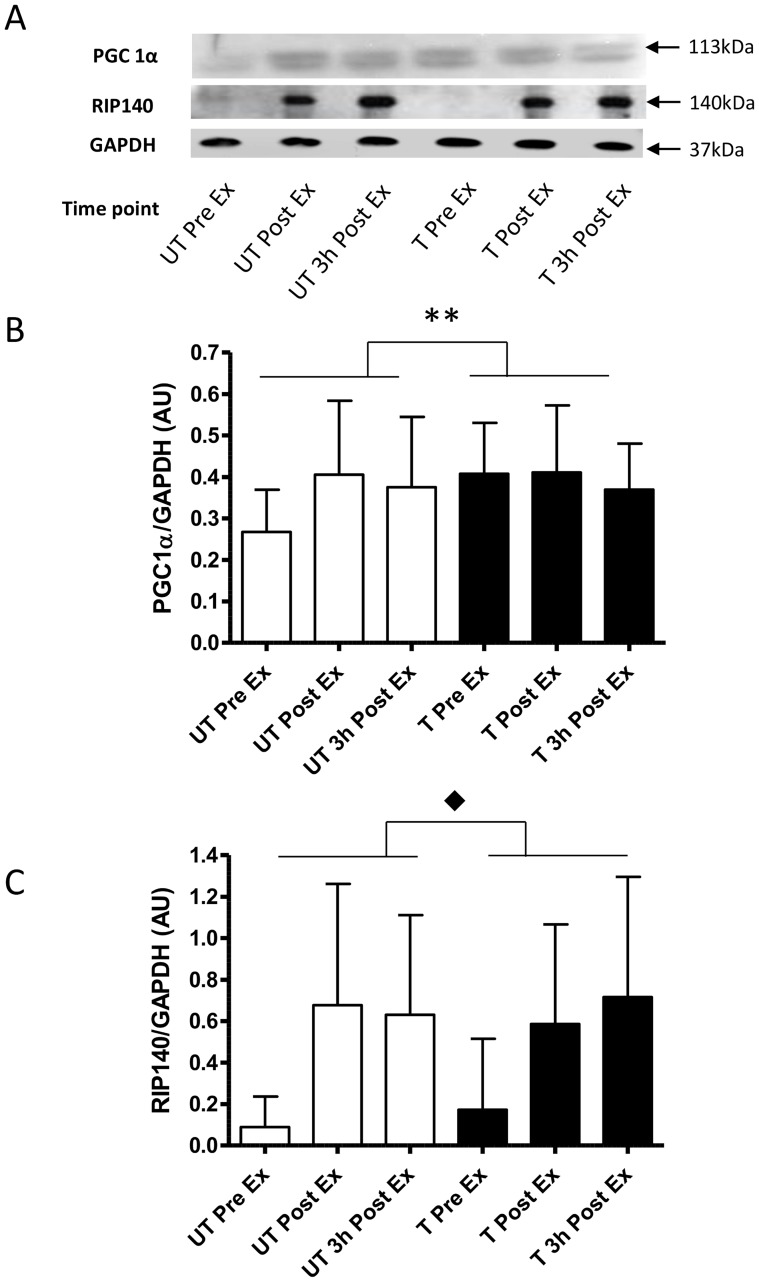
Normalised protein expression changes of the transcription factor co-activator and the co-repressor in response to the same 1 h cycling bout (∼164W or ∼72% VO_2peak_)before and after 10-d of intensified cycle training. A- representative immunoblots, B- peroxisome proliferator-activated receptor γ co-activator -1α (PGC-1α) and C- receptor interacting protein 140 kDa (RIP140). Data are mean ±SD for n = 9. pre-training exercise bout (open bars); post-training exercise bout (closed bars); Pre Ex-resting sample just prior to exercise; Post Ex-sample immediately after exercise; 3 h Post Ex -sample take 3 h after exercise; **- training x time interaction P<0.05; ⧫-main effect of time P<0.01.

Before training, PGC-1α protein expression increased immediately after exercise (1.5±0.6 fold) and remained elevated at this level at 3 h post exercise and before and throughout the post training trial (Training × Time P = 0.05; Figure 4B). The co-repressor RIP140 expression was responsive to acute exercise both before and after training, (Time P = 0.007), where protein expression increased by 7.6±6.5, and 7.1±5.9 fold immediately after exercise and after 3 h of recover pre-training, respectively (Figure 4C). After training the RIP140 protein expression increased by 3.4±3.0 and 4.2±3.4 fold immediately after exercise and 3 h of recovery. Despite the different fold changes in response to acute exercise pre- and post- training the absolute amounts of RIP140 were not different immediately after exercise and recovery (Figure 4C).

#### ETS mitochondrial proteins

**Figure 5:**
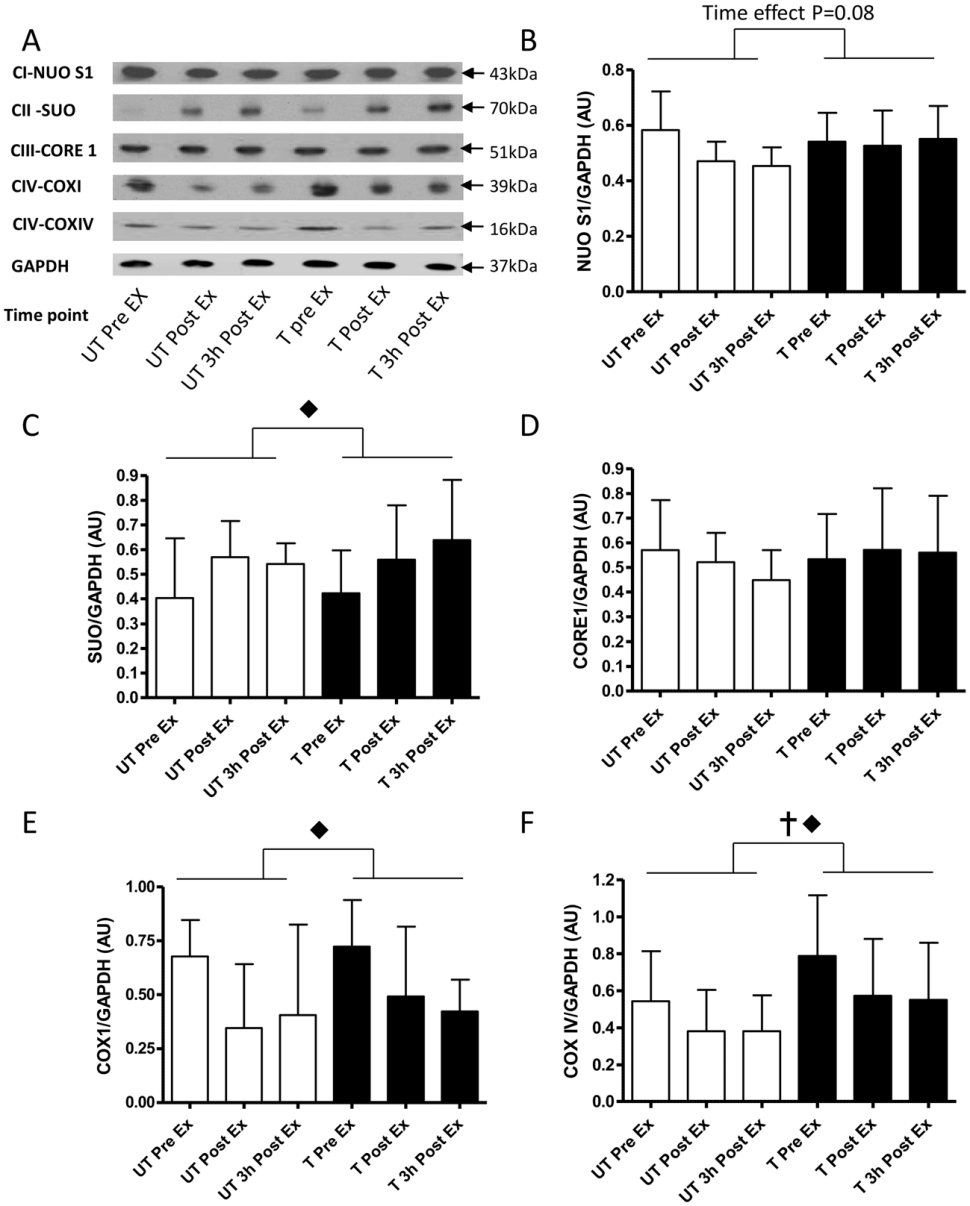
Normalised protein expression changes of representative proteins of the complex I to IV in the mitochondrial electron transport system in response to the same 1 h cycling bout (∼164W or ∼72% VO_2peak_) before and after 10-d of intensified cycle training. A- representative immunoblots, B- NADH-ubiquinol oxidoreductase (NUO-[complex (C)-I]), C- Succinate Ubiqinone Oxyreductase (SUO [C-II]), D- ubiquinol cytochrome c oxidoreductase (Core1 [C-III] ), E- Cytochrome C Oxidase I (COXI [C-IV]) and Cytochrome C Oxidase IV (COXIV [C-IV]). Data are mean ±SD for n = 9. pre-training exercise bout (open bars); post-training exercise bout (closed bars); Pre Ex-resting sample just prior to exercise; Post Ex-sample immediately after exercise; 3 h Post Ex -sample take 3 h after exercise; 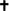
- main effect of training P<0.001; ⧫-main effect of time P<0.01.

Following acute exercise there was reduced expression of two Complex IV proteins COXI (Time P = 0.003; Figure 5E) and COXIV (Time P = 0.001, Training P = 0.004, Figure 5F). Acute exercise, pre-training, reduced COXI and COXIV expression by 49±18% and 30±15% immediately and after 3 h recovery respectively. Similarly, acute exercise after training reduced COXI expression by 36±12% and COXIV by 28±12% immediately and after 3 h recovery. Furthermore COXIV protein was elevated by 1.5±0.9 fold (∼50% increase; P = 0.004) at rest post-training compared to pre-training (Figure 5F). The other mitochondrial ETS protein demonstrating effects of exercise and/or training was Complex II SUO (time P = 0.009; Figure 5C), with acute exercise increasing expression by 1.6±0.9 fold only after 3 h recovery post-training. Complex I NUO expression showed trends for regulation by acute exercise (Time P = 0.08; Figure 5B). On the other hand, Complex III-Core 1 subunit expression was unaffected either acute exercise or short-term training (Figure 5D).

## Discussion

The main finding of this study was that there was a significant attenuation of the increase in PGC1α mRNA expression in response to the same absolute intensity of exercise performed after compared with before 10-days of intensified training. PGC-1α protein expression was increased after the first exercise bout pre-training and was sustained at this level for the post-training exercise bout and recovery. Furthermore, our most surprising finding was acute reduction in the protein expression of COXI and COXIV by acute moderate intensity exercise both before and after exercise training. These data highlight the effects of high intensity short-term exercise training and acute exercise on selected genes and proteins involved in muscle mitochondrial biogenesis and function, suggesting the importance of relative exercise intensity for continued muscle mitochondrial adaptation.

### Regulation of Gene Expression by Acute Exercise before and after Training

#### Transcription factors and co-factors

PGC-1α is considered the master metabolic regulator and promoter of mitochondrial biogenesis [32]. Specifically, exercise bouts including repeated sprint exercise [33], [34], supra maximal intensity interval training (SIT [11], [35]), high intensity interval training (HIIT; [13]) and prolonged submaximal exercise [36]–[40] all increase the gene expression of PGC-1α 2–4 hours after the bout. This gene expression, particularly in response to prolonged submaximal exercise appears to be exercise intensity dependant [36]. In this study the resting muscle gene expression of PGC-1α in response to the training did not change significantly over the course of 10 days of training, a result that concurs with [13]. Our study provides novel insights into the regulation of gene expression in response to acute exercise before and after training, and we clearly demonstrate that PGC-1α gene expression is significantly reduced in response to the same exercise bout post-training (Figure 2). These findings are novel for an acute moderate intensity exercise bout post training, but do concur with [13], who showed an attenuated response of PGC-1α expression to acute high intensity exercise after 6 sessions of HIIT exercise training. This marked reduction in response of PGC-1α mRNA may in fact be due to the lower relative exercise intensity [26] of the post-training exercise bout (72% vs. 64% VO_2peak_ pre- and post-training respectively; [22]).

The mechanism behind reduced PGC-1α mRNA expression has been proposed to result from altered cell signalling, including AMPK, CaMK, Calcinuren, p38 MAPK and mTOR [15]–[17], [41]–[43]. Benziane et al. [22] has previously reported the phosphorylation of these pathways, in these participants, with the attenuation of post exercise AMPK but not CaMKII or p38 signalling demonstrating the strongest association with the reduce PGC-1α mRNA expression post exercise after the training.

The other members of the PGC-1 co-activator family, PGC-1β and PRC, have also been implicated in skeletal muscle adaption to exercise promoting the oxidative muscle phenotype and mitochondrial reticulum expansion through their interaction with NRF transcription factors [44], [45]. This role is further highlighted as we and others have demonstrated PGC-1β and PRC gene expression were responsive to endurance exercise and exercise training [13], [37], [40]. Reduced, PGC-1β gene expression has been linked to muscle mitochondrial biogenesis in a rodent transgenic model [44], which concurs with our data of exercise induced reductions in PGC-1β mRNA pre- and post-training. However, in humans the definitive role of PGC-1β is not clear due to the conflicting results of different exercise and training studies [13], [37], [40]. Mortensen et al. [37], using an acute bout of dynamic knee extensions (3 h) demonstrated decreases in PGC-1β and increases PRC gene expression in responses to exercise that were similar both pre- and post-training (Figure 2). In contrast to other groups [13], [37] we demonstrated no effect of training on PGC-1β gene expression either at rest or in response to exercise after training (Figure 2), which may be explained by the different exercise and training methodologies employed (e.g. duration, intensity and type of exercise) and/or the different timing of the muscle biopsies. Additionally, we demonstrated for the first time an augmented PRC mRNA response to exercise after training, which may indeed serve to promote mitochondrial adaptation in the absence of significant increases of PGC-1α mRNA expression. However, acute increases in mRNA expression nor the exact roles of each of these transcription co-factors in exercise induced mitochondrial adaptation have not been fully elucidated, and warrants further investigation.

The PGC-1 family of co-activators activate the transcription factors Tfam and NRF [46], [47], and have been shown to be effected by acute exercise and exercise training [39], [48]. In this study we observed a trend for increases in Tfam gene expression 3 h after the pre-training exercise bout only, without any changes in NRF2 gene expression (Figure 2). These findings are in contrast to both Pilegaard et al. [39] and Perry et al. [13] who demonstrated increases in NRF2 and Tfam gene expression of ∼1.5 fold 4 h after exercise both before and after training. These contrasting findings again may be explained by different intensities of the exercise bouts and training used. Alternatively, the differences in timing of the biopsy samples between studies may also impact the extent of the mRNA expression post exercise (that is in the current study the biopsies were taken 3 h post while in the other studies they were at 4 and 24 h after exercise). Notwithstanding the limitations, the TFam mRNA expression data are suggestive of a physiologically relevant reduction in mRNA responses to the same acute exercise after training.

#### Mitochondrial genes

The findings that the mRNA for mitochondrial matrix enzymes such as CS and β-HAD were not acutely or chronically regulated by exercise or training was somewhat surprising considering the genes encoding the ETS membrane proteins Cytochrome C and COXVI as well as previous studies [13], [39]. While we cannot rule out that we missed the peak expression of these mRNAs due to the post exercise biopsies being taken too early as discussed previously. However, these data not only suggest that there may be a differential adaptation of mitochondrial matrix and mitochondrial membrane proteins at the mRNA expression level but also suggest that conventional thinking surrounding molecular adaptions to training (i.e. acute and chronic increases in mRNA followed increase in protein expression [13], [49], [50]) may not fully appreciate the complexity of muscle adaptations to exercise as this theory does not currently include regulatory processes such as gene methylation [51], [52] and microRNA’s [40], [53] all of which warrant further investigation.

### Regulation of Protein Abundance by Acute Exercise before and after Training

While gene expression of mitochondrial biogenic factors and proteins are informative about the effects of acute exercise and exercise training, it is ultimately the role of the translated proteins and their post-translational modifications that influence the functionality of the organelle and tissue.

#### Transcription co- factors and co-repressors

The protein abundance of the transcription factor co-activator PGC-1α and its co-repressor RIP140 were investigated due to their apparent opposing function in exercise induced skeletal muscle in mitochondrial adaptation [19], [20]. We noted for the first time that acute exercise increased RIP140 protein expression in skeletal muscle homogenates immediate after exercise and after 3 h of recovery. This is in contrast to other exercise studies where it was demonstrated that RIP140 expression did not change in whole muscle homogenates or nuclear fractions in response to exercise and training [8], [10], [34]. These differing acute exercise responses are difficult to reconcile, but our data are suggestive of acute exercise regulation of protein expression. However, RIP140 abundance was unaffected by training, and has reported by others in rodents and humans [8], [10], [34]. This lack of training induced change in abundance appears to dissociate RIP140 from exercise training induced mitochondrial adaptation. However, the acute exercise responses of RIP140 suggest that it may be important in exercise induced mitochondrial adaptation and that it is via unknown mechanisms *in vivo.* One may speculate that this mechanism is related to post-translational modifications of RIP140 and warrants further *in vitro* mechanistic investigation.

Increased abundance of PGC-1α protein, like the gene expression, has been associated with exercise training induced increases in muscle oxidative capacity [11]–[13]. This study adds further weight to this argument where we demonstrated a ∼50% increase in PGC-1α protein expression after training (Figure 4B). It was surprising that this increase in PGC-1α expression occurred immediately after exercise and was not further increased after the 10-d of training. These data contrasts with the findings of other groups [13], [43], [54] who demonstrated the co-activator protein expression increased 18–24 h after exercise. It is difficult to reconcile these differences between studies as the studies differed in the exercise bouts undertaken, the training interventions and the antibody used. While the specificity of the different PGC-1α antibodies have proved challenging in data interpretation we used the same processes and antibody used by Bartlett et al. [25], who also observed protein bands at ∼113 kDa, providing confidence in our results. Despite the potential antibody limitations, our data and that of others [11]–[13], [43], [54] provide strong evidence that PGC-1α protein abundance increases in response to exercise and training. However, our data raises further questions as to whether a 4-fold increase in PGC-1α after exercise that reaches a 50% higher “steady state” after training is sufficient to induce and maintain mitochondrial adaptation? Furthermore, due to the disproportionately small changes in abundance, we therefore speculate that post-translational modifications of PGC-1α (e.g. phosphorylation, deacetylation etc.) in response to exercise also contributes to exercise induced mitochondrial adaptation. These latter points warrant further mechanistic investigation.

#### ETS proteins

The most important endpoint of mitochondrial biogenesis is increase in abundance of proteins that make up the ETS on the inner mitochondrial membrane. In this study, despite the effectiveness of the training protocol in tightening metabolic control, altering substrate oxidation and maximal aerobic capacity [22], [23] we did not find any training induced changes in representative subunits of C-I to C-III and COXI proteins (Figure 5). We did show that COXIV protein had a training induced change in protein abundance which aligns its increase gene expression and other short-term training studies [7], [9], [10], [13]. Overall, the training induced tightening of metabolic control [22] seems disproportionately large compared to the changes in mitochondrial gene expression and protein abundance after 10-d of training. These data indicate that small changes in mitochondrial protein abundance might alter function, and the mechanisms of this warrant further investigation.

An unexpected finding of this study was the significant reduction in C-IV proteins COXI and COXVI in response to the acute moderate intensity exercise bout before and after training. Acute down regulation (non-significant) in COXIV abundance has been demonstrated after a single HIIT session in untrained humans [13]. Additionally, COX activity has been shown to be down regulated by other acute high-intensity exercise protocols [55], [56]. Our reported loss of COX protein abundance, may in part be explain the decreased enzyme activity, and may be attributed to mitochondrial reticulum remodelling [57], swelling [56] and/or oxidative damage [58]. The functional significance of the exercise induced reductions in C-IV protein abundance are unclear and warrants further investigation.

The main limitation of this study was that we were unable to empirically determine mitochondrial biogenesis by protein synthesis [59] or mitochondrial reticulum expansion using other microscopy or biochemical techniques (eg citrate synthase activity, abundance of voltage dependant anion channels, or mitochondrial DNA copy number) [60] due to study design, tissue sampling procedures and the lack of remaining muscle tissue. However, Larsen et al. [61], indicated that mitochondrial DNA copy number was not indicative of mitochondrial content or function in human muscle. Furthermore, our study design did not include a non-exercise control group or an exercise trial or group undertaking exercise at the same relative intensity exercise post training. The exclusion of the non-exercising control group was based on the powerful repeated measures study design where each participant acts as their own control, the fact that molecular response to exercise are transient [39], [48], molecular adaptations to training in skeletal muscle are the sum of all exercise bouts [13], [49], [50], and training status is an important determinant of molecular responses to exercise [24]. While we cannot definitely exclude the carry over effects of the first pre-training trial, it is highly unlikely that the results in the post-training trial are influenced by this pre-training trial two and a half weeks earlier. PGC-1α mRNA has a half-life in skeletal muscle of approximately 1 hour [62] and its protein abundance in human skeletal muscle returns to baseline two days following a single bout of endurance exercise [63]. Mitochondrial proteins in skeletal muscle have a half-life of several days [64], [65] and are only significantly increased by 3–5 sessions of endurance exercise over a ten day period [13]. Furthermore, the mitochondrial adaptations to several weeks of endurance training are mostly reduced towards baseline by 3 weeks of detraining in humans [66]. Finally, not investigating molecular responses at the same relative exercise intensity post-training, while potentially informative, was due not only to the ethical and practical issues, but the understanding that pre- and post- exercise bouts at the same relative intensity would have participants cycling at ∼189W post-training (vs. ∼164W pre-training; 25W increase in intensity) resulting in different amounts of work being done. Additionally, while the “adaptive” processes in the muscle in our study may be different due to the short-training regimen, there is evidence from a long-term (9-week) training studies in humans that indicate that the active muscle are indeed responding, metabolically (a significant adaptive signal), in an identical manner to the same relative intensity exercise pre- and post-training [67]. Despite these limitations the current data on gene expression and protein abundance changes are suggestive of exercise induced the mitochondrial adaptation.

In conclusion our study provides evidence that 10-d of intensified cycle training reduced PGC-1α mRNA expression, suggestive of reduced mitochondrial biogenesis processes and adaptation in responses to the same (absolute work done) acute bout of endurance exercise in human skeletal muscle. This alteration in molecular programming is potentially due to reduction of relative exercise intensity, and suggests at a molecular level that maintaining relative exercise intensity is important for continued mitochondrial adaptation after as little as 10 consecutive exercise sessions. Additionally, we provide evidence that exercise acutely reduces the protein abundance of COXI and COXIV before and after training, which may impair post exercise mitochondrial function and explain marked and rapid training induced changes in both COXIV mRNA expression and protein abundance. The practical implications of these findings are that we provide evidence that suggests that maintaining absolute exercise intensity even after a short period of endurance training results in a blunting of the molecular programs for mitochondrial reticulum expansion in human skeletal muscle, and may have implications for health and exercise performance.
